# Best practice transfer by public health nurses in Japan: actual conditions and related factors

**DOI:** 10.1186/s12912-024-01800-8

**Published:** 2024-04-22

**Authors:** Mana Fujioka, Reiko Okamoto, Keiko Miyamoto, Keiko Koide, Masako Kageyama, Kazuko Saeki, Kazue Hirokane, Fusami Nagano, Shinji Takemura

**Affiliations:** 1 Former Division of Health Sciences, Osaka University Graduate School of Medicine , Yamadaoka 1-7, 565-0871 Suita-city, Osaka Japan; 2 Division of Health Sciences, Osaka University Graduate School of Medicine, Suita-city, Japan; 3 Faculty of Nursing, Toyama Prefectural University, Nishi-nagae 2-2-78, 930-0975 Toyama-city, Toyama Japan; 4 Faculty of Nursing, Hyogo Medical University, Minatojima 1-3-6, Chuo-ku, 650-8530 Kobe-city, Hyogo Japan; 5 Faculty of Nursing, Fukuyama Heisei University, Kamiiwanari-syoto 117-1, Miyuki-town, 720-0001 Fukuyama-city, Hiroshima Japan; 6 Department of Health Policy and Technology Assessment, National Institute of Public Health, Wako-city-minami 2-3-6, 351-0197 Wako-city, Saitama Japan

**Keywords:** Implementation science, Public health nurse, Best practice, Policy transfer, Actual conditions survey

## Abstract

**Background:**

The workload of public health nurses (PHNs) working for local governments has been increasing as health issues become more diverse and complicated. Even amidst the ongoing administrative and fiscal reforms, there is an urgent need to ensure how effectively and efficiently public health nurses can practice in health service development. The objective of this research was to clarify the actual conditions of best practice transfer (BPT) and its related factors.

**Methods:**

An anonymous postal and self-administered questionnaire survey was conducted among PHNs working at 334 sites, including the local government offices and health centers across Japan, and analysed mainly through logistic regression analysis.

**Results:**

One hundred eighty-five of the 334 institutions (55.4%) agreed to participate, and of the 966 questionnaire forms distributed, 709 forms (73.4%) were collected, of which 702 responses (72.7%) were valid. Although less than half (43.2%) have experience in BPT in health service development, more than 80% are willing to perform going forward. Significant factors for both the group with experience in BPT and the group with willingness to perform include an organizational culture that promotes BPT, as well as multiple elements of the workplace environment and facilitating factors related to knowledge and learning. The experienced group recognised the needs for criteria to evaluate the adaptability of best practice, while the willing group, to evaluate the quality of practice.

**Conclusions:**

Through a nationwide survey, this research elucidated for the first time the actual conditions of BPT by PHNs in Japan and related factors. The results indicated the importance of developing a system to promote BPT at the workplace level, also highlighted the importance for practitioners and experts, including researchers, to work together to develop practical guidelines to ensure evidence-based practices. Urgent actions are needed for the national and local governments to develop a system to promote BPT from diverse perspectives, building on the findings of this research.

**Supplementary Information:**

The online version contains supplementary material available at 10.1186/s12912-024-01800-8.

## Background

Public health nurses working in local governments (PHNs), which account for more than 70% of public health nurses in Japan, are professionals with a separate public role than midwives/nurses under the Act on Public Health Nurses, Midwives, and Nurses [1], and are responsible for supporting populations and developing policies. As health issues become more diverse and complicated, the role of health service development by PHNs is increasing, and their capacity building has become an urgent issue. The Ministry of Health, Labour and Welfare has been working to strengthen the capacity building of PHNs in health service development by increasing the number of credits for relevant subjects in PHN basic education [2] and including their role as a priority in the PHN activity guideline [3]. However, it has reported by Japan Nurses Association there are many PHNs who lack confidence due to the limited opportunities to learn these competencies in pre- and post-graduate education [4], and there is an urgent need for capacity building and system development in accordance with actual conditions.

Amidst the ongoing administrative and fiscal reforms, however, it is difficult to allocate budget to new health services including for PHN activities, making it imperative to find out how to implement health services effectively and efficiently, so evidence-based practice is required in health service development. Since health services implemented by PHNs comprise public health activities with accountability in the public sector, evidence needs always to be integrated in the process of decision-making, planning, implementation and evaluation in health services [5, 6]. Nonetheless, it has been found that many activities are implemented without prior review of evidence [7]. Existing research also indicates that only 50% of the evidence gathered has been applied, and that it will take 17–20 years to put it into practice [8], pointing to the persistent problem of a gap between evidence and practice.

Implementation science is one of the disciplines that have developed to close this gap. It is defined as “the study of methods to promote the adoption and integration of evidence-based practices, interventions, and policies into routine health care and public health settings to improve the impact on population health” [9]. The process by which the knowledge of policies and systems formulated thus far is utilized under different political settings has been studied in Europe as “policy transfer” [10]. Atkins et al. [11] also notes the significance of evidence obtained from practice in the field. Clavier [12] argues that a transferred idea may be embraced as long as it helps solve local problems and points to the necessary contribution of local public health experts as key coordinators in accepting policy transfer.

In Japan, one way to bridge the gap between evidence and practice at the practice level is the best practice transfer in health services (BPT), which is recommended by the government as a way to transfer evidence-based practices, found to be effective in specific geographical areas, to their own local governments. However, at the time of this survey, there were no prior studies on BPT for health service development in Japan, and the Cabinet Office [13] and the National Governors’ Association [14] had only prepared a collection of good practices and published it on their websites. The actual conditions of whether local governments have actually adopted those practices were not clear, have yet to be elucidated as no report has been published on this subject.

We considered that the first step toward that end would be to identify the actual conditions of best practice transfer performed by PHNs at the field level and identifying related factors. It is meaningful to clarify the strategies for capacity building and system development that will contribute to the effective and efficient promotion of best practice transfer by PHNs to improve the population health, using the results of this research as the basic data.

The objective of this research was to elucidate the actual conditions of BPT by PHNs and to analyze the factors related to the conditions.

## Methods

### Definitions

The definition of “best practice” in this research was “any evidence-based, advanced, and effective practices, interventions, and policies established by local governments for their population and communities” operationally in reference to the definition of implementation science [9] described earlier. Practice by PHNs in this paper was the scope of health service development.

The term “transfer” refers to “any activity intended to produce more outcomes through horizontal communication and sharing of the practice across the boundaries of local governments and communities.”

### Setting

This study covered PHNs working at 334 sites, including the government offices of the 47 prefectures and 78 ordinance-designated cities/designated mid-level cities across Japan (as of December 2019), as well as 209 prefectural health centers. We asked six PHNs per site to answer questions. The health centers were randomly sampled with a quota share method. The sample size was determined so that it would exceed the adequate number, approximately 360, calculated assuming a confidence level of 95%, tolerance of 5%, and response rate of 0.5.

### Survey method

This research is a cross-sectional study, an anonymous, self-administered questionnaire survey by mail was conducted. A written request was sent to the principal PHN at each site, along with six questionnaire forms, statements of ethical considerations and return envelopes, to be distributed in a balanced manner in terms of title, years of experience and department. The return envelopes were to be sent by individual PHNs to facilitate collection. The survey was conducted in January 2020.

### Outline of survey

#### Demographics

We asked the respondents about their gender, years of experience as PHN, title and affiliation.

#### Questions on BPT

The questions were drawn up by the research team after a series of deliberations with reference to the Precede–Proceed model [15] to enable actual conditions to be elucidated in a systematic and comprehensive manner. The 17 questions thus determined included: four questions on behavior style and experience impacting on the promotion of BPT; three questions on the workplace environment impacting on BPT; two questions on the knowledge and awareness of BPT as facilitating factors; three questions on enhancement factors, or effective contributors to the promotion of BPT; four questions on realization factors, or enablers of the promotion of BPT; and one question on willingness to perform (Supplementary file 1). Each of these items was asked with two choices, “Experienced, or Not,” and “Existence, or Not.”

#### Analysis method

First, we simply aggregated the answers to the BPT questions in order to grasp the whole picture. Second, statistical tests were conducted to identify the actual conditions of the factors associated with each group of BPT-experienced and BPT-inexperienced but willing to perform. For statistical testing, we used a χ2 test and residual analysis, as well as Fisher’s exact probability test where at least 20.0% of the cells show an expected frequency of under 5. In order to identify factors more strongly associated with each group, then, a logistic regression analysis (stepwise procedure) was conducted with each of these two groups as the dependent variable and the significantly different responses as the independent variables. At this time, years of experience as PHN and affiliation were included as moderating variables to correct for their influence on the results. We used Cramer’s coefficient of association (*r* < 0.7) as a criterion in evaluating multicollinearity between independent variables in the logistic regression analysis. Missing data were excluded from these analyses. We set the statistical significance level at less than 5% and used the statistical software IBM SPSS ver.27.

### Ethical considerations

This research was implemented with the approval of the Ethical Review Board, Osaka University Hospital (Approval No. 19285 dated November 5, 2019). In conducting the survey, we explained the objective and methodology of the research, freedom to cooperate or refuse the survey, personal information protection, and contact information for inquiries, and so forth, in a document attached to the questionnaire form. The respondents gave consent to participation in the survey by ticking the appropriate box in the document and returning it. In order to avoid giving the impression that participation was compulsory, we used return envelopes to collect the completed forms directly from individual respondents, who were supposed to act voluntarily.

## Results

Of the 334 facilities asked to participate, 185 actually cooperated with our research (participation rate of 55.4%). Of the 966 forms distributed, 709 were collected (collection rate of 73.4%), of which 702 contained valid responses (valid response rate of 72.7%). Table 1 shows the demographics of the respondents. 95.7% of the respondents were women. A majority (51.7%) of them had experience of at least 26 years as PHN, followed by 18.8% with 16–25 years, 17.1% with 6–15 years, and 12.4% with 5 years or less. The average number of years of experience was 22.6 (± 11.6). With regard to title, 39.7% had no title while 60.3% were managers. As for affiliation, 24.9% worked at a local government office and 75.1% were stationed at a health center.

**Table 1:** Demographics of the respondents

				*N = 702*
Attributes		Mean (± SD)	*N*	%
Gender	Female		672	95.7
	Male		30	4.3
Years of experience as PHN		22.6 (± 11.6)		
	5 years or less		87	12.4
	6–15 years		120	17.1
	16–25 years		132	18.8
	26 years or more		363	51.7
Title	No title		279	39.7
	Managers		423	60.3
Affiliation	Local government office		175	24.9
	Health center		527	75.1

### Actual conditions of BPT (Table 2)

**Table 2:** Actual conditions of best practice transfer (BPT)

					*N = 702*
Factors				*n*	%
Behavior style and experience	Experience of BPT in service development		Experienced	303	43.2
	Experience of difficulty in service development		Experienced	635	90.5
	Expectations for best practice in service development		Existence	633	90.2
	Sources of best practice		Websites of national and local governments	570	81.2
			Trainers and lecturers	415	59.1
			Professional publications	396	56.4
			Other websites	378	53.8
			Inquiry to local governments nearby	244	34.8
			Supervisors	166	23.6
			Colleagues	123	17.5
			Japan Medical Abstracts and other article search tools	78	11.1
			Inquiries to universities	25	3.6
			Other	24	3.4
Workplace environment	Opportunities for learning about BPT		Existence	247	35.2
	Desire to learn about BPT		Existence	603	85.9
	Organizational culture that promotes BPT		Existence	364	51.9
Facilitating factors	Knowledge of BPT		Consulted websites and relevant materials	292	41.6
			Only heard of it	235	33.5
	Awareness of the importance of BPT		Existence	671	95.6
Enhancement factors	Recognition of benefit of BPT for local residents		Existence	653	93.0
	Expectations for reduced burden by adopting best practice		Existence	617	87.9
	Support and systems required to promote BPT	Supporter	Expert support	492	70.1
			Supervisor’s support	412	58.7
			Head’s support	248	35.3
		Material	Case studies available online	379	54.0
			Published case studies	293	41.7
			Introduction video	97	13.8
		Budget	Production development budget	580	82.6
			Inspection budget	234	33.3
		Information	Information exchange sessions	374	53.3
			Mailing list for distributing information	177	25.2
			Periodical magazine	166	23.6
		Training	Skill training	404	57.5
			Methodological guidelines	372	53.0
			Review meeting	237	33.8
		Other		16	2.3
Realization factors	Awareness of the need for criteria to evaluate the quality of practice		Existence	456	65.0
	Criteria to evaluate the quality of practice evaluation		Clarification of outcome evaluation	571	81.3
			Clarification of implementation process	520	74.1
			Reaction of local residents	468	66.7
			Clarification of structure including budgeting, staffing and collaborating systems	403	57.4
			Evaluation by others	183	26.1
			Enthusiasm/thoughts of those involved	68	9.7
			Other	15	2.1
	Awareness of the need for criteria to evaluate the applicability of practice to the local community		Existence	487	69.4
	Criteria to evaluate the applicability of best practice		The needs and demands of local residents	537	76.5
			Suitability/affinity with the local community	530	75.5
			Procedural convenience	400	57.0
			Flexibility to allow improvements	367	52.3
			Clarification of success factors and how to meet challenges	364	51.9
			Ease of explaining to stakeholders	263	37.5
			Enjoyment of the program	187	26.6
			Follow-up from the originator	79	11.3
			Other	14	2.0
Willingness to perform	Willingness to perform BPT going forward		Existence	586	83.5

Values for existence or nonexistence responses are given for the existence group only

#### Behavior style and experience

To the question on “experience of BPT in health service development,” 43.2% answered that they had such experience. 90.5% had “experience of difficulty in health service development,” whereas 90.2% had “expectations for best practice in health service development.” Asked about “sources of best practice,” most cited websites of national and local governments (81.2%), followed by trainers and lecturers (59.1%) and professional publications (56.4%). Japan Medical Abstracts and other article search tools (11.1%) and inquiries to universities (3.6%) were not popular options.

#### Workplace environment

35.2% had “opportunities for learning about BPT,” while 85.9% had a “desire to learn about BPT.” 51.9% enjoyed an “organizational culture that promotes BPT.”

#### Facilitating factors

Asked about “knowledge of BPT,” 41.6% answered that they had consulted websites and relevant materials, while 33.5% had only heard of it. 95.6% were aware of the “importance of BPT.”

#### Enhancement factors

93.0% were aware of the “benefit of BPT for local residents,” while 87.9% had “expectations for reduced burden by adopting best practice.” Asked about “support and systems required to promote BPT,” 82.6% cited production development budget, followed by expert support (70.1%). A majority of respondents also cited supervisor’s support (58.7%), skill training (57.5%), case studies available online (54.0%), information exchange sessions (53.3%) and methodological guidelines (53.0%).

#### Realization factors

65.0% were aware of the “need for criteria to evaluate the quality of practice.” Asked about necessary “criteria to evaluate the quality of practice evaluation,” most of the respondents cited clarification of outcome evaluation (81.3%), followed by clarification of implementation process (74.1%), reaction of local residents (66.7%) and clarification of structure including budgeting, staffing and collaborating systems (57.4%). 69.4% were aware of the “need for criteria to evaluate the applicability of practice to the local community.” Asked about necessary “criteria to evaluate the applicability of best practice,” a substantial number of respondents cited the needs and demands of local residents (76.5%) and suitability/affinity with the local community (75.5%). A majority of the respondents also cited procedural convenience (57.0%), flexibility to allow improvements (52.3%) and clarification of success factors and how to meet challenges (51.9%).

#### Willingness to perform

83.5% of the respondents had “willingness to perform BPT going forward.”

### Relationship between BPT experienced/nonexperienced group (Table 3)

**Table 3:** Relationship between BPT experienced/nonexperienced, and existence/nonexistence of willingness to perform BPT going forward in the inexperienced group

				*N* = 702								*N* = 399					
Word Factors				Experience of BPT in service development								[Breakdown of Inexperienced group] Willingness to perform BPT going forward in the group without experience of BPT					
				Experienced group (n = 303)			Inexperienced group (n = 399)					Existence group (n = 302)		Nonexistence group (n = 97)			
				*n*	%		*n*	%		*P*		*n*	%	*n*	%	*P*	
Behavior style and experience	Experience of difficulty in service development		Experienced	279	92.7		356	93.4		0.702		278	96.9	78	83.0	< 0.001	*
	Expectations for best practice in service development		Existence	288	96.0		345	89.1		0.001	*	276	94.8	69	71.9	< 0.001	*
	Sources of best practice		Websites of national and local governments	267	88.1		303	75.9		< 0.001	*	240	79.5	63	64.9	0.004	*
			Trainers and lecturers	202	66.7		213	53.4		< 0.001	*	167	55.3	46	47.4	0.176	
			Professional publications	191	63.0		205	51.4		< 0.001	*	160	53.0	45	46.4	0.259	
			Other websites	160	52.8		218	54.6		0.630		173	57.3	45	46.4	0.061	
			Inquiry to local governments nearby	122	40.3		122	30.6		0.008	*	101	33.4	21	21.6	0.028	*
			Supervisors	66	21.8		100	25.1		0.311		87	28.8	13	13.4	0.002	*
			Colleagues	68	17.0		55	18.2		0.702		55	18.2	13	13.4	0.273	
			Japan Medical Abstracts and other article search tools	42	13.9		36	9.0		0.043	*	28	9.3	8	8.2	0.759	
			Inquiries to universities	17	5.6		8	2.0		0.011	*	6	2.0	2	2.1	1.000	^a)^
			Other	20	6.6		4	1.0		< 0.001	*	1	0.3	3	3.1	0.046	*^a)^
Workplace environment	Opportunities for learning about BPT		Existence	154	50.8		93	23.3		< 0.001	*	79	26.2	14	14.4	0.017	*
	Desire to learn about BPT		Existence	270	89.1		333	83.5		0.033	*	271	89.7	62	63.9	< 0.001	*
	Organizational culture that promotes BPT		Existence	226	74.6		138	34.6		< 0.001	*	122	40.4	16	16.5	< 0.001	*
Facilitating factors	Knowledge of BPT		Consulted websites and relevant materials	183	60.4		109	27.3		< 0.001	*^b)^	87	28.8	22	22.7	0.101	
			Only heard of it	86	28.4		149	37.3				117	38.7	32	33.0		
	Awareness of the importance of BPT		Existence	295	97.4		376	94.2		0.046	*	298	98.7	78	80.4	< 0.001	*
Enhancement factors	Recognition of benefit of BPT for local residents		Existence	279	92.7		356	93.4		0.702		292	96.7	78	80.4	< 0.001	*
	Expectations for reduced burden by adopting best practice		Existence	280	92.4		337	84.5		0.001	*	277	91.7	60	61.9	< 0.001	*
	Support and systems required to promote BPT		Expert support	215	71.0		277	69.4		0.660		215	71.2	62	63.9	0.176	
		Supporter	Supervisor’s support	177	58.4		235	58.9		0.898		197	65.2	38	39.2	< 0.001	*
			Head's support	112	37.0		136	34.1		0.429		103	34.1	33	34.0	0.988	
			Case studies available online	169	55.8		210	52.6		0.408		172	57.0	38	39.2	0.002	*
		Material	Published case studies	128	42.2		165	41.4		0.813		135	44.7	30	30.9	0.017	*
			Introduction video	42	13.9		55	13.8		0.977		45	14.9	10	10.3	0.254	
		Budget	Production development budget	263	86.8		317	79.4		0.011	*	246	81.5	71	73.2	0.080	
			Inspection budget	112	37.0		122	30.6		0.075		101	33.4	21	21.6	0.028	*
			Information exchange sessions	157	51.8		217	54.4		0.499		176	58.3	41	42.3	0.006	*
		Information	Mailing list for distributing information	91	30.0		86	21.6		0.010	*	70	23.2	16	16.5	0.164	
			Periodical magazine	78	25.7		88	22.1		0.255		67	22.2	21	21.6	0.912	
			Skill training	166	54.8		238	59.6		0.197		190	62.9	48	49.5	0.019	*
		Training	Methodological guidelines	165	54.5		207	51.9		0.498		161	53.3	46	47.4	0.313	
			Review meeting	103	34.0		134	33.6		0.910		109	36.1	25	25.8	0.061	
		Other		10	3.3		6	1.5		0.114		3	1.0	3	3.1	0.157	^a)^
Realization factors	Awareness of the need for criteria to evaluate the quality of practice		Existence	197	65.0		259	64.9		0.977		217	71.9	42	43.3	< 0.001	*
	Criteria to evaluate the quality of practice evaluation		Clarification of outcome evaluation	256	84.5		315	78.9		0.062		248	82.1	67	69.1	0.006	*
			Clarification of implementation process	229	75.6		291	72.9		0.428		232	76.8	59	60.8	0.002	*
			Reaction of local residents	213	70.3		255	63.9		0.075		202	66.9	53	54.6	0.029	*
			Clarification of structure including budgeting, staffing and collaborating systems	191	63.0		212	53.1		0.009	*	163	54.0	49	50.5	0.553	
			Evaluation by others	89	29.4		94	23.6		0.082		74	24.5	20	20.6	0.433	
			Enthusiasm/thoughts of those involved	32	10.6		36	9.0		0.495		29	9.6	7	7.2	0.475	
			Other	8	2.6		7	1.8		0.421		3	1.0	4	4.1	0.063	^a)^
	Awareness of the need for criteria to evaluate the applicability of practice to the local community		Existence	219	72.3		268	67.2		0.146		224	74.2	44	45.4	< 0.001	*
	Criteria to evaluate the applicability of best practice		The needs and demands of local residents	235	77.6		302	75.7		0.563		231	76.5	71	73.2	0.511	
			Suitability/affinity with the local community	236	77.9		294	73.7		0.200		225	74.5	69	71.1	0.512	
			Procedural convenience	168	55.4		232	58.1		0.474		185	61.3	47	48.5	0.026	*
			Flexibility to allow improvements	175	57.8		192	48.1		0.011	*	153	50.7	39	40.2	0.073	
			Clarification of success factors and how to meet challenges	178	58.7		186	46.6		0.001	*	146	48.3	40	41.2	0.222	
			Ease of explaining to stakeholders	112	37.0		151	37.8		0.811		119	39.4	32	33.0	0.257	
			Enjoyment of the program	86	28.4		101	25.3		0.362		79	26.2	22	22.7	0.493	
			Follow-up from the originator	38	12.5		41	10.3		0.347		31	10.3	10	10.3	0.990	
			Other	9	3.0		5	1.3		0.107		2	0.7	3	3.1	0.095	^a)^
Willingness to perform	Willingness to perform BPT going forward		Existence	284	93.7		302	75.7		< 0.001	*						

Chi-square test: *P*<0.05 *, Values for existence or nonexistence responses are given for the existence group only

a) is Fisher's direct probability test

b) is residual analysis +: indicates that the number of observed frequencies is higher than the expected frequency (*P* < 0.05)

-: indicates that the observed frequency is lower than the expected frequency (*P* < 0.05)

#### With or without experience of BPT

303 respondents (43.2%) had “experience of BPT in health service development, while 399 respondents did not (56.8%).

Answers given by a significantly higher number of respondents in the “experienced” group (*P* < 0.05) to the question about “sources of best practice” in the behavior style/experience category included websites of national and local governments, trainers and lecturers, professional publications, inquiry to local governments nearby, Japan Medical Abstracts and other article search tools, inquiry to universities and “other.” In the workplace environment and facilitating factor categories, the experienced group showed a higher percentage of affirmative answers than the “inexperienced” group across the board, including the questions on “opportunities for learning about BPT,” “willingness to learn about BPT,” “organizational culture that promotes BPT,” “knowledge of BPT” and “awareness of the importance of BPT” (*P* < 0.05). As regards enhancement factors, the experienced group had a significantly higher share of those with “expectations for reduced burden by adopting best practice,” as well as those citing health service development budget and mailing list for distributing information as “support and systems required to promote BPT” (*P* < 0.05). Concerning realization factors, the experienced group had a larger share of respondents who cited clarification of structure including budgeting, staffing and collaborating systems as necessary “criteria to evaluate the quality of practice,” as well as those who included flexibility to allow improvements and clarification of success factors and how to meet challenges in the necessary “criteria to evaluate the applicability of best practice” (*P* < 0.05). As for willingness to perform, those with “willingness to perform BPT going forward” had a larger share in the experienced group (*P* < 0.05).

#### Willingness to perform BPT going forward in the group without experience of BPT

Of the 399 respondents in the group without “experience of BPT in health service development,” 302 (75.7%) showed “willingness to perform BPT going forward,” while 97 (24.3%) did not.

The “willing” group significantly exceeded the “unwilling” group (*P* < 0.05) in the share of those who had “experience of difficulty in health service development” and “expectations for best practice in health service development” regarding behavior style/experience, as well as those who cited websites of national and local governments, inquiry to local government nearby, supervisors and “other” as “sources of best practice.” With regard to the workplace environment and facilitating factors, a larger share of respondents in the willing group than in the unwilling group had “opportunities for learning about BPT,” “desire to learn about BPT,” “organizational culture that promotes BPT,” and “awareness of the importance of BPT” (*P* < 0.05). In terms of enhancement factors, the willing group included a significantly higher percentage of respondents with “awareness of the benefit of BPT for local residents” and “expectations for reduced burden by adopting best practice,” as well as those who cited supervisor’s support, case studies available online, published case studies, inspection budget, information exchange sessions and skill training as “support and systems required to promote BPT” (*P* < 0.05). As for realization factors, the willing group had a significantly higher percentage of those with “awareness of the need for criteria to evaluate the quality of practice” and “awareness of the need for criteria to evaluate the applicability of practice to the local community,” those who included clarification of outcome evaluation (quantitative change), clarification of implementation process (PDCA/procedure) and reaction of local residents in the necessary “criteria to evaluate the quality of practice,” and those who cited procedural convenience among the necessary “criteria to evaluate the applicability of best practice” (*P* < 0.05).

### Logistic regression analysis

#### With or without experience of BPT (Table 4)

**Table 4:** Related factors of experience of BPT in service development (logistic regression analysis)

			*N*=687	
Independent variable	Odds ratio	95% confidence interval	*P*-value	
F: Knowledge of BPT: Consulted websites and relevant materials	4.23	2.53–7.07	< 0.001	*
W: Organizational culture that promotes BPT	4.14	2.85–6.02	< 0.001	*
P: Willingness to perform BPT going forward	2.78	1.53–5.07	0.001	*
F: Knowledge of BPT: Only heard of it	2.07	1.23–3.48	0.006	*
W: Opportunities for learning about BPT	1.71	1.16–2.51	0.006	*
R: Criteria to evaluate the applicability of best practice: Clarification of success factors and how to meet challenges	1.48	1.03–2.12	0.035	*

Adjustment variables: 4 groups of years of experience as PHN and 2 groups of affiliation; *P* < 0.05 *

(Years of experience: 5 years or less = 0 as standard. Affiliation: Local government office = 1, Health center = 0)

Category code for each factor: W; Workplace environment, F; Facilitating factors, R; Realization factors,

P; Willingness to perform

We performed a logistic regression analysis defining “experience of BPT in health service development” as a dependent variable, and all other answers to the questions related thereto as independent variables. Significant relationships (*P*<0.05) were observed, in order of odds ratio (OR), with: “knowledge of BPT: consulting websites and relevant materials” (OR: 4.23, 95%CI: 2.53–7.07); “organizational culture that promotes BPT” (OR: 4.14, 95%CI: 2.85–6.02); “willingness to perform BPT going forward” (OR: 2.78, 95%CI: 1.53–5.07); “knowledge of BPT: have only heard of it” (OR: 2.07, 95%CI: 1.23–3.48), “opportunities for learning about BPT” (OR: 1.71, 95%CI: 1.16–2.51); “criteria to evaluate the applicability of best practice: clarification of success factors and how to meet challenges” (OR: 1.48, 95%CI: 1.03–2.12).

#### Willingness to perform BPT going forward in the group without experience of BPT (Table 5)

**Table 5:** Related factors of willingness to perform BPT going forward in the group without experience of BPT (logistic regression analysis)

			*N*=379	
Independent variable	Odds ratio	95% confidence interval	*P*-value	
F: Awareness of the importance of BPT	11.57	3.14–42.60	< 0.001	*
B: Experience of difficulty in project development	5.72	1.92–17.06	0.002	*
E: Expectations for reduced burden by adopting best practice	3.41	1.58–7.36	0.002	*
B: Expectations for best practice in service development	2.98	1.22–7.29	0.017	*
E: Support and systems required to promote BPT: Supervisor’s support	2.82	1.56–5.07	0.001	*
W: Organizational culture that promotes BPT	2.57	1.30–5.09	0.007	*
W: Desire to learn about BPT	2.34	1.16–4.75	0.018	*
B: Sources of best practice: Inquiry to local governments nearby	2.04	1.03–4.03	0.040	*
R: Awareness of the need for criteria to evaluate the quality of practice	1.92	1.05–3.49	0.034	*

Adjustment variables: 4 groups of years of experience as PHN and 2 groups of affiliation; *P* < 0.05 *

(Years of experience: 5 years or less = 0 as standard. Affiliation: Local government office = 1, Health center = 0)

Category code for each factor: B; Behavior style and experience, W; Workplace environment, F; Facilitating factors,

E; Enhancement factors, R; Realization factors

We performed a logistic regression analysis defining “willingness to perform BPT going forward” as a dependent variable, and all other answers to the questions related thereto as independent variables. Significant relationships (*P*<0.05) were observed, in order of odds ratio (OR), with: “awareness of the importance of BPT” (OR: 11.57, 95%CI: 3.14–42.6); “experience of difficulty in health service development” (OR: 5.72, 95%CI: 1.92–17.06); “expectations for reduced burden by adopting best practice” (OR: 3.41, 95%CI: 1.58–7.36); “expectations for best practice as a model of health service development” (OR: 2.98, 95%CI: 1.22–7.29); “support and systems required to promote BPT: supervisor’s support” (OR: 2.82, 95%CI: 1.56–5.07); “organizational culture that promotes BPT” (OR: 2.57, 95%CI: 1.30–5.09); “desire to learn about BPT” (OR: 2.34, 95%CI: 1.16–4.75); “sources of best practice: inquiry to local governments nearby” (OR: 2.04, 95%CI: 1.03–4.03); “awareness of the need for criteria to evaluate the quality of practice” (OR: 1.92, 95%CI: 1.05–3.49).

## Discussion

This research is unique and innovative in that it elucidates the actual conditions of BPT by PHNs in Japan and related factors for the first time through a survey on a national scale. We received 702 valid responses from a parent population of some 6,000. With a response rate of over 10%, we believe that an adequate sample size was secured. The fact that over half of the respondents were managers with experience of at least 26 years means that the samples included a substantial number of PHNs with a sufficient career in PHN practice and policy management, effectively ensuring the collection of data suited to the research.

### Actual conditions of BPT

#### Behavior style, experience and willingness to perform

Less than half of the PHNs had experience of BPT in health service development, revealing delays in the implementation of BPT at the field level. It is also true, however, that over 90% of the PHNs recognized difficulties in health service development and had expectations for best practice to serve as a model, and that more than 80% indicated a willingness to perform BPT in developing a health service going forward. This finding highlights the need for urgent development of a system for promoting BPT.

We also found that the PHNs were using easily accessible information as 80% cited websites of national and local governments, and 60% trainers and lectures, as sources of best practice. Although half of the respondents also cited professional publications, they rarely used article search tools (11.1%) or made inquiries to universities (3.6%), implying challenges for implementing evidence-based practice.

#### Workplace environment and facilitating factors

We found that PHNs had a substantial interest in, awareness of the importance of, and willingness to learn about BPT to gain maximum impact by effectively utilizing limited resources, as they were aware that the Cabinet Office and the Association of Prefectural Governors are promoting BPT (75.1%), believed in its importance (95.6%), and wanted to learn about it, given the opportunity (85.9%). However, not many of them had an opportunity to learn about BPT in health service development (35.2%) or enjoyed a workplace environment that promoted BPT (51.9%), revealing inadequacies in the training system or enabling culture at the workplace.

#### Enhancement factors

We found that PHNs were highly aware that applying proven best practice to the local community serves the interest of the residents (93.0%) and that applicable best practice will reduce their own burden (87.9%).

Many respondents cited health service development budget (82.6%) and expert support (70.1%) as the support and systems required to promote BPT, which indicates that PHNs prioritized financial resources necessary for developing or revising relevant practice and collaboration with experts to ensure the quality of knowledge and skills as foundations for promoting BPT. Answers given by a majority of respondents suggested the need for technical support and systems including training and guidelines, as well as physical and informational support and systems including online case studies and information exchange sessions. Such support and systems will have to be developed in order to promote BPT going forward.

#### Realization factors

The necessity of criteria to evaluate the quality of practice and its applicability to the local community was demonstrated as two out of three respondents answered that such criteria would help promote BPT.

As necessary criteria for evaluating the quality of practice, many cited the clarification of outcome evaluation (81.3%), implementation process (74.1%) and structure (57.4%), attesting to the relevance of the framework for evaluating the quality of healthcare proposed by Donabedian [16]. We also found that PHNs put priority on the benefit of users participating in the health service, as many answered that the reaction of local residents (66.7%) would be an appropriate criterion.

PHNs’ focus on the local community was also demonstrated by the fact that three out of four respondents included the needs/demands of local residents and suitability/affinity with the local community among the necessary criteria for evaluating the applicability of best practice. Three other answers given by a majority of the respondents were related to the convenience of the health service for healthcare providers.

Practical criteria, including those noted above, will need to be developed in order to promote BPT going forward.

### Factors related to BPT

#### Characteristics of factors related to BPT

The expertise and awareness/behavior that were significantly more common to the experienced group than the inexperienced group spanned all areas, ranging from behavior style/experience to the workplace environment, facilitating factors, enhancement factors, realization factors and willingness to perform. Within the inexperienced group, the expertise and awareness/behavior of the willing group also exceeded those of the unwilling group across the board. These results indicate the need to consider all those factors in a comprehensive manner in order to promote BPT in the future. In particular, the important items for promoting BPT were considered in the logistic regression analysis to be the organizational culture that promotes BPT in the workplace environment that were raised in both Tables 4 and 5, items related to BPT learning opportunities and desire, and criteria to evaluate the quality/applicability of best practice. All of this suggests that having evidence in health service development and criteria for assessing its applicability, as well as collaboration at the workplace level, are very important for promoting BPT. The importance of the content of these items is also supported by their inclusion in the consolidated framework for implementation research (CFIR) [17, 18], the most commonly used D&I research, in items “implementation climate: tension for change, learning climate, etc.”, which correspond to inner setting, and ”evidence strength & quality, adaptabillity”, which correspond to intervention characteristics.

#### Factors promoting BPT in health service development

Logistic regression analysis suggested that the group of BPT-experienced and the group of BPT-inexperienced but willing to perform have different learning support strategies for capacity building. Although the odds ratios of items corresponding to facilitating factors were highest in both groups, knowledge of BPT was most important in the experienced group, while awareness of the importance of BPT was most important in the inexperienced group. In addition, none of the items corresponding to behavioral style/experience and enhancement factors were listed by the experienced group, while five items were listed by the inexperienced group. This suggests that it is effective to provide learning support for the inexperienced group to make them aware of the importance of BPT, to enable them to make use of their own experience of difficulties in health service development, to reduce their burden by using successful practices as models, and to establish a system in which they can receive support from their supervisors in this process. We think that these results suggest what kind of support is needed to promote BPT in the future.

### Policy recommendations

BPT is the process of implementing and disseminating proven best practice. The promotion of BPT is expected to serve as a method of evidence-based practice that can produce effects most efficiently. We recommend that national and local governments proceed with the following policies, as our findings described above have elucidated the key points in developing a system to promote BPT. These include: (1) seeking the leadership of, and collaboration with experienced PHNs and supervisors to nurture an enabling organizational culture for BPT; and to that end (2) establishing a system to ensure opportunities for learning and information exchange to obtain knowledge about BPT, heighten awareness of its importance and enhance willingness to perform; (3) working with researchers and other experts to develop criteria for evaluating the quality of practice and its applicability to the local community, as well as implementation guidelines in order to develop EBP; (4) building a website and collecting case studies to facilitate the search for best practice; and (5) supporting to secure budget at the local government level to advance those activities.

In its Policy Brief [19], the World Health Organization lists cost effectiveness, adaptation of values/norms/needs, simple to understand and use, opportunity for trial, having observable benefits and adaptability, among others, as requirements for transferring service and policy innovations in health systems. A previous study [20] reported that a strong institutional capacity and the existence of research partnerships are two of the factors facilitating knowledge transfer among policymakers and researchers. We believe that those findings are relevant in promoting actions (1) and (5) or in developing the criteria and guidelines for action (3). Elsewhere, findings might be leveraged from the education, business and implementation science sectors where expertise has been accumulated concerning the transfer of best practice. Our research will help provide increasingly diverse perspectives for promoting BPT, after identifying requirements to develop a system to promote BPT.

In addition, there are currently many D&I models that contribute to improving the quality of health services [21]. In the future, to develop a capacity building system for PHNs, we may be able to draw on the consolidated framework for implementation research (CFIR) [18], the model for adaptation design and impact (MADI) [22], and the existing training course “Putting Public Health Evidence in Action” [23], which include items similar to those identified in this survey. I think it would also be useful to consider a Japanese version of the training course by modifying them to fit the Japanese context identified in this survey results.

### Limitations

There are two limitations of the present research. First, being a cross-sectional study, our research was limited to analyzing relevance and therefore could not identify causalities. Second, as the first nationwide survey on the actual conditions of BPT by PHNs, this research only covered PHNs stationed at the government offices and health centers of prefectures and major cities, who are often involved in health service development, and therefore cannot elucidate the actual conditions of PHNs working in other municipalities. As the first survey of its kind, we also had to cover the whole public health sector. Going forward, however, criteria for evaluating the quality of best practice and methods of BPT will need to be considered for each area and type of health service.

## Conclusions

Through a nationwide survey, this research elucidated for the first time the actual conditions of BPT by PHNs in Japan and related factors. Although less than half (43.2%) have experience in BPT in health service development, more than 80% are willing to perform going forward. Significant factors for both the group with experience in BPT and the group with willingness to perform include an organizational culture that promotes BPT, as well as multiple elements of the workplace environment and facilitating factors related to knowledge and learning, which indicates the importance of developing a system to promote BPT at the workplace level. The need for criteria to evaluate the adaptability of best practice regarding the experienced group, and to evaluate the quality of practice concerning the willing group, also highlights the importance for practitioners and experts, including researchers, to work together to develop practical guidelines. Urgent actions are needed to develop a system to promote BPT from diverse perspectives, building on the findings of this research.

### Electronic supplementary material

Below is the link to the electronic supplementary material.

Supplementary Material 1

## Data Availability

The datasets analyzed during the current study are not publicly available due to need further analysis but are available from the corresponding author on reasonable request.

## Abbreviations

PHNs: Public health nurses
BPT: Best practice transfer
