# Supplementary material for: Best practice transfer by public health nurses in Japan: actual conditions and related factors
Source: BMC Nurs. 2024 Apr 22;23:253. doi: 10.1186/s12912-024-01800-8 (PMC11034165; doi:10.1186/s12912-024-01800-8)
Supplement: Supplementary file 1 — Supplementary Material 1 [file 12912_2024_1800_MOESM1_ESM.pdf]

Questionnaire for actual conditions of best practice transfer (BPT)

Circle the answer that applies to you.

Factors				1	2
Behavior style and experience	Experience of BPT in service development		Experienced	Yes	No
	Experience of difficulty in service development		Experienced	Yes	No
	Expectations for best practice in service development		Existence	Yes	No
	Sources of best practice		Websites of national and local governments	Yes	No
			Trainers and lecturers	Yes	No
			Professional publications	Yes	No
			Other websites	Yes	No
			Inquiry to local governments nearby	Yes	No
			Supervisors	Yes	No
			Colleagues	Yes	No
			Japan Medical Abstracts and other article search tools	Yes	No
			Inquiries to universities	Yes	No
			Other	Yes	No
Workplace environment	Opportunities for learning about BPT		Existence	Yes	No
	Desire to learn about BPT		Existence	Yes	No
	Organizational culture that promotes BPT		Existence	Yes	No
Facilitating factors	Knowledge of BPT		Consulted websites and relevant materials	Yes	No
			Only heard of it	Yes	No
	Awareness of the importance of BPT		Existence	Yes	No
Enhancement factors	Recognition of benefit of BPT for local residents		Existence	Yes	No
	Expectations for reduced burden by adopting best practice		Existence	Yes	No
	Support and systems required to promote BPT	Supporter	Expert support	Yes	No
			Supervisor's support	Yes	No
			Head's support	Yes	No
		Material	Case studies available online	Yes	No
			Published case studies	Yes	No
			Introduction video	Yes	No
		Budget	Production development budget	Yes	No
			Inspection budget	Yes	No
		Information	Information exchange sessions	Yes	No
			Mailing list for distributing information	Yes	No
			Periodical magazine	Yes	No
		Training	Skill training	Yes	No
			Methodological guidelines	Yes	No
			Review meeting	Yes	No
		Other		Yes	No
Realization factors	Awareness of the need for criteria to evaluate the quality of practice		Existence	Yes	No
	Criteria to evaluate the quality of practice evaluation		Clarification of outcome evaluation	Yes	No
			Clarification of implementation process	Yes	No
			Reaction of local residents	Yes	No
			Clarification of structure including budgeting, staffing and collaborating systems	Yes	No
			Evaluation by others	Yes	No
			Enthusiasm/thoughts of those involved	Yes	No
			Other	Yes	No
	Awareness of the need for criteria to evaluate the applicability of practice to the local community		Existence	Yes	No
	Criteria to evaluate the applicability of best practice		The needs and demands of local residents	Yes	No
			Suitability/affinity with the local community	Yes	No
			Procedural convenience	Yes	No
			Flexibility to allow improvements	Yes	No
			Clarification of success factors and how to meet challenges	Yes	No
			Ease of explaining to stakeholders	Yes	No
			Enjoyment of the program	Yes	No
			Follow-up from the originator	Yes	No
			Other	Yes	No
Willingness to perform	Willingness to perform BPT going forward		Existence	Yes	No
